# Clinical Performance Comparison of LMA Protector™ Cuff Pilot™ and LMA Supreme™ When Used in Anesthetized, Non-paralyzed Patients

**DOI:** 10.7759/cureus.23176

**Published:** 2022-03-15

**Authors:** Weng Ken Chan, Chian Yong Liu

**Affiliations:** 1 Anaesthesia, Universiti Kebangsaan Malaysia, Kuala Lumpur, MYS; 2 Anaesthesiology and Intensive Care, Hospital Umum Sarawak, Kuching, MYS

**Keywords:** airway management, oropharyngeal leak pressure, olp, lma supreme, lma protector, laryngeal mask

## Abstract

Introduction

The advancement of supraglottic airways (SGAs) has eased airway management, especially for anesthetists. There were functional improvements implemented to the newer SGA. We aim to assess the clinical performance of laryngeal mask airway (LMA) Protector™ Cuff Pilot™ (Teleflex Inc., Wayne, Pennsylvania, USA) against LMA Supreme™ (Teleflex Inc., Wayne, Pennsylvania, USA), in terms of oropharyngeal leak pressure (OLP), successful insertion attempts, mean insertion time, ease of gastric tube insertion, laryngeal view, and incidence of sore throat among anesthetized, non-paralyzed patients undergoing general anesthesia.

Methods

In this prospective single-blinded study, 60 patients were randomized to use either LMA Protector™ Cuff Pilot™ or LMA Supreme™. Both groups received standard monitoring and induction regimes. Post-insertion, a bronchoscope was used to verify its position. A gastric tube was inserted and OLP was measured. Patients were assessed during the post-operative period for sore throats.

Results

LMA Protector™ Cuff Pilot™ was comparable to LMA Supreme™ in terms of mean OLP (30.72±8.60 vs 27.23±8.09 cmH_2_O, *P *= 0.114), first successful attempt (*P* = 0.312), mean insertion time (27.72±9.45 vs 24.37±6.46 seconds, *P *= 0.116), and grade 1 laryngeal view (51.7% vs 36.7%, *P *= 0.244). At first attempt, LMA Protector™ Cuff Pilot™ had a lower success rate of gastric tube insertion than LMA Supreme™ (55.17% vs 96.67%, *P *<0.001). The incidence of the blood-stained device and sore throat post-operatively were comparable between the two groups.

Conclusion

LMA Protector™ Cuff Pilot™ was comparable to LMA Supreme™ in terms of overall clinical performance, except for the first successful gastric tube insertion. Improvements should be made to the gastric channel for easier gastric tube insertion in the LMA Protector™ Cuff Pilot™.

## Introduction

This article was previously presented as a poster at the Evidence Based Perioperative Medicine-Asia Congress (EBPOM-ASIA) 2019 on November 1-3, 2019.

The inception of supraglottic airways (SGAs) has revolutionized the anesthetist’s airway armamentarium. It was invented by Dr. Archie Ian Jeremy Brain in 1982 and was commercially available in 1987 [1]. It has the advantage of avoiding endotracheal intubation, a shorter insertion time, lower incidence of post-operative pharyngeal pain, and better hemodynamic stability during induction and emergence [2]. An ideal SGA placement should provide sufficient perilaryngeal seal to allow adequate ventilation of the lungs with minimal pharyngeal mucosa injury. Secondly, it must also be able to protect the airway from gastric content aspiration or provide early detection of gastric content regurgitation. Thirdly, an ideal SGA placement should be able to facilitate the intubation process via its airway tract.

Supraglottic airways can be classified based on device generation [3], cuff design, reusability, and sealing mechanism [4]. SGA is commonly classified based on device generation as introduced by Cook and Howes [3]. It is divided into three generations: the first generation, which is a simple airway device such as classic laryngeal mask airway (cLMA). Second generation SGAs, such as ProSeal™ (Teleflex Inc., Wayne, Pennsylvania, USA) laryngeal mask airway (LMA), include an additional gastric channel that aims to reduce risk of gastric aspiration. A recent controversial new term, third generation, was introduced for commercial purposes to indicate the presence of self-energizing sealing cuffs such as Baska [5]. Other methods of classification can be whether it is cuffed such as cLMA and ProSeal™ LMA or uncuffed such as i-gel, Baska, and streamlined liner of the pharynx airway (SLIPA). It can also be divided into single-used devices such as LMA Supreme™ (Teleflex Inc., Wayne, Pennsylvania, USA) or reusable such as ProSeal™ LMA. Supraglottic airways can also be classified based on their sealing mechanism, such as perilaryngeal seals like cLMA or base of pharyngeal seals like SLIPA.

LMA Supreme™ is a single-use, second-generation SGA, which utilizes perilaryngeal seal by Teleflex® that was introduced in 2005. It is made of polyvinyl chloride (PVC). It has the advantage of an anatomically shaped airway tube and the presence of an integral bite block and a drain tube to facilitate placement of the gastric tube [6]. According to the manufacturer, it is a high volume low pressure cuff that can generate a high seal pressure of up to 37 cmH_2_O [7].

LMA Protector™ Cuff Pilot™ (Teleflex Inc., Wayne, Pennsylvania, USA) is a single-use SGA with a perilaryngeal seal and is the latest second generation SGA from Teleflex® introduced in 2015. It is made of silicon and is both latex and phthalate free, as contrary to LMA Supreme™. It is similar to LMA Supreme™ in that it has a dynamic curve that conforms to the anatomical contour of the pharynx, hence allowing rapid insertion. The LMA Protector™ Cuff Pilot™ also has an integral bite block and additional dual gastric access. Furthermore, it has an integrated cuff pressure monitor to ensure the SGA is optimally inflated [8].

The common complications of SGA are malposition, sore throat, dysphagia, and laryngeal nerve injury [9]. An instruction leaflet for LMA Supreme™ advised a maximum cuff volume of 20 ml, 30 ml, and 40 ml of air for sizes 3, 4, and 5, respectively, to achieved desired intra-cuff pressure. It recommends a maximum intra-cuff pressure limit of 60 cmH_2_O [6]. However, inflating the maximum recommended volume of the cuff often results in intra-cuff pressure higher than 60 cmH_2_O [10,11]. Saracoglu reported that professional experience does not associate with optimal inflation of cuff pressure without measuring it [12]. Hence, this calls for the need to introduce a cuff manometer into our routine anesthetic practice.

The new LMA Protector™ Cuff Pilot™ was designed to reduce the risk of overinflation. It has a cuff pilot valve to allow the user to monitor the intra-cuff pressure of the SGA through a visual means that is color-coded. Optimal intra-cuff pressure of 40-60 cmH_2_O will place the cuff pilot valve in the green zone, whereas underinflating and overinflating will place it either in the yellow or red zone, respectively. As it is made of silicone, it also offers more flexibility and is potentially less traumatic to the pharyngeal mucosa than LMA Supreme™ [13].

## Materials and methods

This prospective, single blinded, randomized controlled trial has been approved by the Research Committee of the Department of Anesthesiology and Intensive Care, Universiti Kebangsaan Malaysia Medical Centre (UKMMC) and the Medical Research and Ethics Committee, UKMMC (No. FF-2018-130). This trial has also been registered with ClinicalTrials.gov (NCT 03984032) and was funded via a research grant by UKMMC.

Patients who were planned for surgery under general anesthesia were screened for eligibility according to predetermined inclusion and exclusion criteria. They were invited to participate in this study after being counseled. A patient information sheet (PIS) either in Malay or English was given to them. Written informed consent was obtained from patients who agreed to participate in this study.

This study was conducted in the operation theaters (OTs) of UKMMC from July 2018 to March 2019. We enrolled 60 patients aged 18-65 years who were planned for any surgery requiring general anesthesia without muscle relaxation. We excluded patients with body mass index (BMI) >35 kg m^−^^2^, anticipated difficult intubation patients (simplified airway risk Index score ≥4) [14], and patients with increased risks of gastric aspiration.

**Figure 1:**
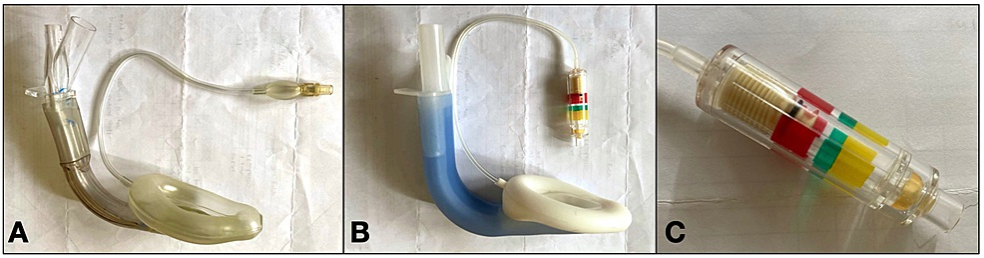
Supraglottic airway. (A) LMA supreme™. (B) LMA protector™ Cuff Pilot™. (C) Cuff pilot™ with colour zones indicating corresponding intra-cuff pressure.

They were randomized into either group P or group S according to the online random sequence generator (https://www.random.org/sequences/). Patients in group P had LMA Protector™ Cuff Pilot™ inserted during general anesthesia induction, whereas patients in group S used LMA Supreme™ (Figure 1). The selection of SGA size was done according to the manufacturer’s recommendation based on patients' weight. In the OT, patients were placed in a supine position on a head rest. Standard monitoring consisting of pulse oximetry, non-invasive blood pressure (NIBP), three-lead electrocardiogram (ECG), and end-tidal gas monitoring was used.

Both groups received similar induction regimes consisting of preoxygenation to achieve an end-tidal fractional oxygen (ETO_2_) concentration >0.85; intravenous (IV) fentanyl 1-2 mcg kg^−^^1^; and IV propofol 1.5-2.5 mg kg^−^^1^. Anesthesia was maintained with sevoflurane at a minimum alveolar concentration (MAC) of 0.8-1.2 via manual mask ventilation with the adjustable pressure limiting (APL) valve closed at <20 cmH_2_O. Each SGA was fully deflated, and its posterior surface was lubricated with water-based gel adequately prior to placement. SGA was inserted once the patient's pupils were centered and there was an absence of motor response to jaw thrust. Both LMA Protector™ Cuff Pilot™ and LMA Supreme™ were inserted using the single-handed rotational technique in the semi-sniffing position as recommended by the manufacturer. No neuromuscular blockade was administered. This study was performed by a single operator who had prior training on both SGA using mannequins and clinical experience on at least six patients.

After insertion, the cuff in group P was insufflated with air till the cuff pilot valve indicator reached the center of the green zone, corresponding to an estimated cuff pressure of 40-60 cmH_2_O. In group S, the cuff was insufflated with air in accordance to the manufacturer’s recommendation to 60 cmH_2_O via a hand-held analog cuff pressure gauge (VBM, Medizintechnik GmbH, Germany). The insertion time, which was defined as the duration from picking up the study device to the presence of capnography tracing, was recorded. The number of attempts was also recorded. Patients who required more than three attempts were considered failed attempts and were subsequently managed appropriately by the attending anesthetist. The position of the SGA in relation to the laryngeal inlet was verified by passing an intubating bronchoscope to a position just proximal to the end of the SGA. The laryngeal view obtained at this point was scored according to Keller et al.: grade 1, clear view of the vocal cords; grade 2, view of the arytenoids only; grade 3, view of the epiglottis only; and grade 4, no laryngeal structures visible [15]. A size 12-F gastric tube was lubricated at the distal tip with water-based lubricant prior to insertion into the gastric channel of SGA. The number of gastric tube insertion attempts was recorded. Correct placement of the gastric tube was determined by the detection of injected air through epigastric auscultation. Oropharyngeal leak pressure (OLP) assessment was performed by setting the adjustable pressure limiting (APL) valve of the circle system at 40 cmH_2_O with a fresh gas flow of 3 l min^−^^1^. The OLP was determined by observing the value of airway pressure at equilibrium and with a stethoscope placed over the mouth to listen to the presence of the first audible noise. For safety reasons, the maximum allowable OLP was 40 cmH_2_O. Intraoperatively, anesthesia was maintained with sevoflurane at a MAC of 0.8 to 1.2 in a mixture of 50% oxygen and 50% medical air with a total flow of 2 l min^−^​​​​​​​^1^. Subsequent anesthetic management, including analgesia and anti-emetic, was in accordance to the discretion of the anesthetist-in-charge. SGA was removed once the patient was awake and obeying simple commands. The presence of blood stains over the SGA was recorded. Participants were followed up on the hospital ward till six hours post-operatively to assess for presence of sore throat.

We hypothesized that LMA Protector™ Cuff Pilot™ has a similar OLP to LMA Supreme™. The primary objective was to compare the OLP between LMA Protector™ Cuff Pilot™ and LMA Supreme™. From the literature reviews on both SGA, we needed 26 participants in each group to detect the difference of 4.5 cmH_2_O (20.7 vs 25.2 cmH_2_O) [16] with a standard deviation of 5.7 at 80% power and an alpha value of 0.05 using Power and Sample Size Calculation version 3.1.2 (Department of Biostatistics, Vanderbilt University School of Medicine, Nashville, USA) [17]. With the anticipation of a 15% dropout rate, we enrolled a total of 60 participants. Our secondary objectives were to compare mean time to insertion and attempts, ease of gastric tube insertion, laryngeal view, incidences of sore throat, and the presence of bloodstain among both SGA. All data were entered and analyzed in Statistical Package for Social Science (SPSS) version 20.0 (IBM Corp., Armonk, New York, USA) using independent *t*-test, Pearson Chi-square, and Fisher's Exact test as appropriate. A value of P <0.05 was considered statistically significant.

## Results

**Figure 2:**
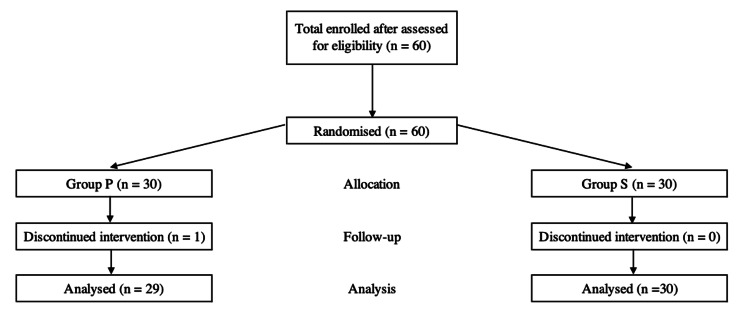
Flow diagram of patient recruitment.

A total of 60 patients were recruited, with 30 patients in each group. There were 29 completed data for LMA Protector™ Cuff Pilot™ (group P) arm as there was a patient who had failed insertion after three attempts, which was subsequently managed successfully by inserting LMA ProSeal™ in a single attempt (Figure 2). Demographic characteristics of patients and intraoperative characteristics between both groups were comparable except for the duration of surgery (Table 1 and Table 2).

**Table 1:** Demographic characteristics of patients. ^a^Independent t test. ^b^Pearson Chi-square. Data were presented as a mean standard deviation (SD) or as a number (%).

Variables	Group P (n = 30)	Group S (n = 30)	P value
Age (years)	39.63 ± 14.43	41.17 ± 13.42	0.672^a^
Weight (kg)	65.70 ± 16.76	67.31 ± 13.94	0.688^a^
Gender			0.795^b^
Male	14 (46.67)	13 (43.33)	
Female	16 (53.33)	17 (56.67)	

**Table 2:** Intraoperative characteristics. ^a^Pearson Chi-square, ^b^Independent t-test, *P = 0.011 when comparing the duration of surgery among both groups. Data were presented as a mean standard deviation (SD) or as a number (%).

Variables	Group P (n = 29)	Group S (n = 30)	P value
LMA sizes			0.127^a^
3	8 (27.59)	3 (10.00)	
4	10 (34.48)	17 (56.67)	
5	11 (37.93)	10 (33.33)	
Duration of surgery (min)	67 ± 28	96 ± 52	0.011^b,^*

Group P has a comparable first successful attempt rate with group S (P = 0.222). The mean insertion time for both groups was also comparable (27.72±9.45 vs 24.37±6.46 seconds, P = 0.116). OLP was determined from 29 patients in group P and 30 patients in group S. The mean OLP of group P was comparable with that of group S (30.72±8.60 vs 27.23±8.09 cmH_2_O, P = 0.114). Primary and secondary outcomes are shown in Table 3.

**Table 3:** Primary and secondary outcome data. ^a^Independent t test, ^b^Pearson Chi-square test, ^c^Grading according to Keller [15], *P < 0.001 when comparing the first successful gastric tube insertion attempt between both SGA. Data were presented as a mean standard deviation (SD) or as a number (%).

Variables	Group P (n = 29)	Group S (n = 30)	P value
OLP (cmH_2_O)	30.72 ± 8.60	27.23 ± 8.09	0.114^a^
Insertion time (seconds)	27.72 ± 9.45	24.37 ± 6.46	0.116^a^
Successful LMA insertion			
First successful attempt	21 (72.41)	25 (83.33)	0.312^b^
>1 Attempt	8 (27.59)	5 (16.67)	
Laryngeal view^c^			
Grade 1	15 (51.72)	11 (36.67)	0.244^b^
Grade 2-4	14 (48.28)	19 (63.33)	
Gastric tube insertion			
One attempt	16 (55.17)	29 (96.67)	<0.001^b,^*
Two attempts	1 (3.45)	-	
Abandoned	12 (41.38)	1 (3.33)	
Blood stain			
Present	18 (62.07)	13 (43.33)	0.150^b^
Absent	11 (37.93)	17 (56.67)	
Sore throat			
Yes	10 (34.48)	8 (26.67)	0.514^b^
No	19 (65.52)	22 (73.33)	

Both group P and group S recorded 51.72% and 36.67% of laryngeal view grade 1, respectively (P = 0.244). Group P has a lower success rate of gastric tube insertion (P < 0.001) and is more difficult to insert as compared to group S. Group P and group S recorded an incidence of 62.07% and 43.33%, respectively, in regard to bloodstains observed on SGA after removal (P = 0.150). We found that patients in group P had an incidence of sore throat of 34.48% as compared to group S of 26.67% after six hours post-operatively. However, this was comparable in terms of the incidence of post-operative sore throat (P = 0.514). Further sub-analysis within LMA Protector™ Cuff Pilot™ and LMA Supreme™ also did not show any significant association between first successful attempt and operation >1 hour with the incidence of sore throat post-operatively.

## Discussion

Oropharyngeal leak pressure is an indirect measure of SGA’s efficacy in providing ventilation and protection against aspiration. A higher OLP provides a better perilaryngeal seal, hence being able to withstand higher peak airway pressure before gases leak out and contribute to stomach insufflation. From our study, the mean OLP of LMA Protector™ Cuff Pilot™ was 30.72±8.60 cmH_2_O. This is consistent with Zaballos [18], who reported a median OLP of 31 (interquartile range (IQR): 26-36)) cmH_2_O and Chang [19], who reported a mean OLP of 30.8±6.6 cmH_2_O in a group of paralyzed patients. Only one study reported a higher median OLP of 34 (IQR: 28.4-35) cmH_2_O [20]. When a comparison was made between LMA Protector™ Cuff Pilot™ and LMA Supreme™, Moser [21] was able to demonstrate a significant difference between the OLP of both SGA (30.9±7.4 cmH_2_O vs 25.6±4.4 cmH_2_O, P < 0.001). This difference may be attributed to their difference in the mode of anesthesia, which was induction with a higher dose of fentanyl and propofol, as well as maintenance of anesthesia using propofol and remifentanil infusion.

Although the design of LMA Supreme™ is smaller and more rigid as compared to LMA Protector™ Cuff Pilot™, both of them had comparable insertion times (P = 0.116) and successful insertion attempts (P = 0.222). This was coherent with Moser's finding on LMA Protector™ Cuff Pilot™ and LMA Supreme™ (insertion time: 29.4±2.4 vs 28.7±2.2 seconds, respectively, P = 0.162; insertion attempts: P = 1.000) [21].

During confirmation of SGA placement via a bronchoscope, we noted that both LMA Protector™ Cuff Pilot™ and LMA Supreme™ had comparable grade 1 laryngeal view (P = 0.244). Even though both SGA have comparable laryngeal views, LMA Protector™ Cuff Pilot™ offers the extra advantage of a larger airway lumen internal diameter, which can be used to facilitate endotracheal tube (ETT) insertion should the need arise.

We reported a lower first-attempt successful gastric tube insertion in the LMA Protector™ Cuff Pilot™ group as compared to the LMA Supreme™ group (55.17% vs 96.67%, P < 0.001). In the study by Moser [21], they also reported a better first-attempt successful gastric tube insertion rate clinically among the LMA Supreme™ group as compared to the LMA Protector™ Cuff Pilot™ group (94% vs 85%) (P = 0.317). The study done by Zaballos [18] concurred on the difficulty in gastric tube insertion as well (easy insertion for 81% of participants). This finding may be explained by the SGA’s material, design, and gastric channel outlet in relation to the upper esophagus of the patient. LMA Supreme™ is made of PVC, hence it has less resistance as compared to LMA Protector™ Cuff Pilot™, which is made of silicon. In our clinical practice, a large amount of water-soluble lubricant had to be applied to lubricate the LMA Protector™ Cuff Pilot™’s gastric channel in order to facilitate smoother gastric tube passage. LMA Protector™ Cuff Pilot™ also has a reservoir in its gastric outlet (31-42 ml depending on size), which we thought was a potential space for coiling of the gastric tube. We suggest that modifications should be made to better facilitate gastric tube insertion. Alternatively, a gastric tube should be inserted with the tip protruding out from the gastric channel outlet prior to SGA insertion.

We found that there is a higher incidence of bloodstain present on the posterior surface of LMA Protector™ Cuff Pilot™ than LMA Supreme™ (62.07% vs 43.33%, P = 0.150). Moser [21] also reported a higher rate of blood staining in the LMA Protector™ Cuff Pilot™ group as compared with LMA Supreme™ (19% vs 2%). We also found that patients in the LMA Protector™ Cuff Pilot™ group had a higher incidence of sore throat after six hours post-operatively as compared to the LMA Supreme™ group (34.48% vs 26.67%). In the study by Zaballos [18], they reported an incidence of bloodstains and sore throats of 25% and 24%, respectively. In our study, we did not find any association between insertion attempts with the incidence of sore throat and operation duration with the incidence of sore throat among the two SGA groups.

One of the limitations of our study was that we did not grade the amount of blood staining on SGA and the severity of sore throat. The sample size in this study was adequately powered to investigate the primary objective. However, larger sample size will be needed for secondary objectives. In view of the small sample size, there was bias in terms of operation duration for both groups (P = 0.011). This bias may affect the finding with regard to the incidence of sore throat. We also suggest other parameters such as height, BMI, and thyromental distance [22] in relation to SGA sizes should be investigated.

## Conclusions

In conclusion, we found that LMA Protector™ Cuff Pilot™ was comparable to LMA Supreme™ in terms of overall clinical performance, except for the first successful gastric tube insertion. LMA Protector™ Cuff Pilot™ also has an additional advantage as an intubating conduit due to its wider airway channel as compared to LMA Supreme™. This can facilitate the passage of an adult-sized tracheal tube. However, we suggest that the gastric channel should be modified to improve gastric tube first-pass success.
